# Effect of Combined Application of Wood Vinegar Solution and Biochar on Saline Soil Properties and Cotton Stress Tolerance

**DOI:** 10.3390/plants13172427

**Published:** 2024-08-30

**Authors:** Liu Yang, Guangmu Tang, Wanli Xu, Yunshu Zhang, Songrui Ning, Pujia Yu, Jie Zhu, Qingsong Wu, Peng Yu

**Affiliations:** 1 College of Resources and Environment, Xinjiang Agricultural University, Urumqi 830052, China; yangliu201875@163.com (L.Y.); zhujie190520@163.com (J.Z.); 2 Institute of Soil Fertiliser and Agricultural Water Conservation, Xinjiang Academy of Agricultural Sciences/Key Laboratory of Saline and Alkaline Land Improvement and Utilisation (Arid and Semi-Arid Zone Saline and Alkaline Land), Ministry of Agriculture and Rural Affairs, Urumqi 830091, China; wlxu2005@163.com (W.X.); zhangyunshu@xaas.ac.cn (Y.Z.); 3 State Key Laboratory of Eco-Hydraulus in Northwest Arid Region of China, Xi’an University of Technology, Xi’an 710048, China; ningsongrui@163.com; 4 School of Geographical Sciences, Southwest University, Chongqing 400715, China; yupujia@swu.edu.cn; 5 Student Work Department, Hulunbuir Vocational Technical College, Hulunbuir 021000, China; anjiben@sina.cn; 6 Department of Chemical Engineering, Hulunbuir Vocational Technical College, Hulunbuir 021000, China; 18047016465@163.com

**Keywords:** wood vinegar solution, biochar, single application, combined application, enzyme activity, resistance

## Abstract

Biomass pyrolysis by-products, such as biochar (BC) and wood vinegar (WV), are widely used as soil conditioners and efficiency enhancers in agriculture. A pot experiment was conducted to examine the effects of WV, both alone and in combination with BC, on soil properties in mildly saline soil and on cotton stress tolerance. The results demonstrated that BC and WV application, either individually or together, increased soil nutrient content. The combined application was more effective than the individual applications, resulting in a 5.18–20.12% increase in organic matter, a 2.65–15.04% increase in hydrolysable nitrogen, a 2.23–58.05% increase in effective phosphorus, and a 2.71–29.38% increase in quick-acting potassium. Additionally, the combined application of WV and BC led to greater improvements in cotton plant height, net photosynthetic rate (Pn), leaf nitrate reductase (NR), superoxide dismutase (SOD), and catalase (CAT) activities compared to the application of BC or WV alone. The enhancements in this study varied across different parameters. Plant height showed an increase of 14.32–21.90%. Net photosynthetic rate improved by 13.56–17.60%. Leaf nitrate reductase increased by 5.47–37.79%. Superoxide dismutase and catalase showed improvements of 5.82–64.95% and 10.36–71.40%, respectively (*p* < 0.05). Moreover, the combined treatment outperformed the individual applications of WV and BC, resulting in a significant decrease in MDA levels by 2.47–51.72% over the experimental period. This combined treatment ultimately enhanced cotton stress tolerance. Using the entropy weight method to analyze the results, it was concluded that the combined application of WV and BC could enhance soil properties in mildly saline soils, increase cotton resistance, and hold significant potential for widespread application.

## 1. Introduction

Biochar (BC) and wood vinegar (WV) are high-value added products with certain characteristics and functions formed during the pyrolysis process of biomass [1]. BC is considered to be a soil amendment with good application prospects due to its rich porosity, functional groups [2], high specific surface area, and strong adsorption capacity [3]. Some studies have shown that BC application can improve the physicochemical and biological properties of soil [4], increase soil water content [5] and nutrient utilization efficiency, change the structure of the microbial community [6], activate the antioxidant capacity of the plant [4], and enhance crop stress tolerance, thus achieving an increase in yield. However, the addition of BC is not beneficial to crop growth under all conditions, which mainly depend on the characteristics of BC, preparation conditions, soil type [7], etc. BC also increases soil pH and total water-soluble salts due to the combination of the carbonates contained in BC and the release of inorganic ions in the soil, which exacerbates the process of soil salinity [8] and limits the application of BC for improving saline-alkaline soil. Therefore, finding effective ways to suppress the increasing trend of soil salinization caused by BC application is necessary to mitigate the potential risks of BC in saline-alkaline soil improvement.

WV, also known as pyromellitic acid, mainly contains more than 200 organic compounds, such as water, organic acids, alcohols, phenols, aldehydes, and ketones, as well as trace elements such as Fe and Zn [9]. WV, as a multifunctional soil amendment, can reduce soil salinity and pH, inhibit ammonium loss, mitigate N_2_O and CH_4_ emissions [10], regulate the bacterial community, inactivate phytopathogenic bacteria [8], promote crop growth, and increase crop yield [11]. However, the low nutrient content of the WV substrate limits its supply of nutrients to crops; therefore, it is more important to investigate the effects of the combined application of WV and other exogenous substances on improving crop yield and quality. Some studies have shown that the combined application of WV and BC can reduce the abundance of soil antibiotic resistance genes [12], and can also improve the fruit yield and nutritional quality of peanuts [13] and blueberry [14] in saline soils.

According to statistics, Xinjiang’s cotton production in 2023 was 5,112,000 t, accounting for 90.1% of the national total [15], which holds a pivotal position in the national cotton industry pattern. Aridity, scarce precipitation, and high evaporation are the main climatic characteristics of the Xinjiang region, leading to the continuous accumulation of soluble salts on the surface; this results in increased soil pH and water-soluble salt content [16], thereby restricting cotton growth and yield enhancement. The improvement and utilization of saline soils are of great significance to the development of agricultural production in Xinjiang, especially for the cotton industry. Physical, chemical, biological, and hydraulic engineering improvement measures [17] are the main existing methods for saline-alkaline land improvement, which have the characteristics of improving soil structure and environment [18], low risk of contamination, alleviating salt stress, a rapid effect, and a noticeable effect; however, they also have the disadvantages of requiring large amounts of engineering, high cost, serious waste of water resources, susceptibility to salt return, reliance on single technologies, and the introduction of new salt ions [19,20]. WV and BC are derived from the pyrolysis of biomass, such as agricultural and forestry waste straw, and contain substances such as acetic acid, biochar, phenols, and ketones that improve salinity, increase crop resistance, improve soil structure, and increase the efficiency of fertilizers and medicines; moreover, their joint application can achieve synergistic effects. Meanwhile, WV and BC are of great significance in promoting biomass energy sourcing and high-value utilization.

Currently, it has been demonstrated that BC can increase antioxidant enzyme activities and reduce the MDA content of apple seedling leaves [21], and that, as an energy input into the soil ecosystem, it can significantly improve the stochastic selection of soil bacterial communities and reduce the dominant effect of salinity on soil bacterial communities [22]. WV can significantly increase the number of fungi and bacteria in alkaline soils, and it can effectively activate the activities of soil enzymes in 3 days. Soil sucrase, urease, alkaline phosphatase, and dehydrogenase activities were increased to different degrees [23]. However, most of the previous studies focused on WV and BC alone, failing to fully consider the advantages of their combined application. Some studies have shown that the combined application of BC and humic acid is more effective than BC alone in reducing soil pH and EC [14], and that the paired application of BC and nitrogen fertilizers stimulates soil enzyme activities and root plasticity [24]. For this reason, in this study, the biomass pyrolysis products of WV and BC were used as soil conditioner materials. Through potting experiments, we investigated the effects of the combined application of WV and BC on the physicochemical properties of soil in mildly saline conditions and assessed the effects of the combined application of WV and BC on the resilience of cotton plants in order to provide a theoretical basis for the improvement and utilization of WV and BC in saline and alkaline soils.

## 2. Materials and Methods

### 2.1. Test Site and Test Soil

The experiment was located within the experimental site of the Soil Fertiliser and Agricultural Water Conservation Research Institute of Xinjiang Academy of Agricultural Sciences, Shaybak District, Urumqi City, Xinjiang Uygur Autonomous Region (43°48′47″ N, 87°34′11″ E). The site is located in the hinterland of the Asian-European continent, at the northern foot of the middle part of the Tien Shan mountain range and the southern edge of the Junggar Basin. It has a mid-temperate continental arid climate, with a multi-year average temperature of 8.03 °C, an average annual precipitation of 194 mm, and an average annual sunshine duration of 2768 h. The soil was collected from Xinjiang Uygur.

The test soil was collected from the Tsukuba Experimental Station, Changji Hui Autonomous Prefecture, Xinjiang Uygur Autonomous Region (44°06′33″ N, 87°20′01″ E), and was randomly collected from the 0 to 40 cm plow layer of the saline farmland, naturally air-dried, and then passed through a 2 mm sieve for further use. The physicochemical properties of the test soil were as follows: hydrolyzable nitrogen 84.5 mg·kg^−1^, effective phosphorus 29.8 mg·kg^−1^, quick-acting potassium 268 mg·kg^−1^, organic matter 10.6 g·kg^−1^, pH 8.98, indicating that it was a mildly alkaline soil with an alkalinity of 5.84%. During the test period, the average temperature in Urumqi was 23.43 °C, precipitation was 0.53 mm, average soil temperature was 22.01 °C, and average humidity was 72.19%.

### 2.2. Physicochemical Properties of BC and WV for Testing

The test BC and WV were made from cotton straw, which was cracked at 450 °C for 2 h under oxygen isolation. WV was the clarified supernatant of the pyrolysis condensate, which was brownish, pH 2.78, with 80% water content, and comprised approximately 11% acid, 3% esters, 1% alcohols, 1% ketones, and 0.7% phenols, along with other organic matter such as furans. BC was the solid material resulting from the pyrolysis of cotton straw, which was crushed and passed through a 2 mm sieve, and had the following characteristics: pH 10.12, EC 12.21 mS·cm^−1^, effective phosphorus 1004.8 mg·kg^−1^, all carbon 669.49 g·kg^−1^, all nitrogen 3.63 g·kg^−1^, and all phosphorus 3.25 g·kg^−1^.

### 2.3. Experimental Design

The experiment was conducted using plastic pots with the dimensions 16.5 × 16.5 × 17.5 cm. Each pot had a soil bulk density of 1.35 g·cm^−3^, a soil depth of 15 cm, and contained 5.5 kg of soil. The experimental design included a total of 7 treatments, each replicated 3 times. The treatments were as follows: CK, which served as the control with no wood vinegar (WV) or biochar (BC) added; BC1, where 1.5% BC (based on dry soil mass), equivalent to 82.5 g of biochar per 5.5 kg of soil, was thoroughly mixed with the soil before sowing; BC2, where 3% BC, or 165 g of biochar per 5.5 kg of soil, was similarly mixed with the soil before sowing; WV1, where 1.5% WV (*w*/*w*) was applied through drip irrigation in six applications over 30 days after seedling emergence, when seedlings had 3–4 cotyledons; WV2, where 3% WV (*w*/*w*) was applied in six similar drip irrigation applications over the same period; WB1, where 1.5% BC was added in six applications before sowing; and WB2, where 3% BC was added in six applications before sowing. The trial was conducted outdoors under natural conditions, and irrigation water was dosed at the upper limit of 70% of the soil water holding capacity (mineralization of the irrigation water was 378 mg·L^−1^).

Fertilizer application rates for the pot experiment were as follows: N at 300 kg·ha^−1^, P_2_O_5_ at 150 kg·ha^−1^, K_2_O at 120 kg·ha^−1^, 100% basal; urea (China National Petroleum Corporation, Beijing, China, N:46%) at 40% basal and 20% as a follow-up at bud stage. Soil samples from the 0–10 cm surface layer, plant samples, and leaf photosynthetic rates were collected at 30 days, 60 days, and 90 days after seedling emergence for the determination of soil pH, total soluble salt, salt ions, plant enzyme activity, and root vigor; soil enzyme activity indexes were destructively collected at 90 days after seedling emergence for a single determination of soil samples.

### 2.4. Sample Analyses

Soil pH, EC, total soluble salts, salt ions, hydrolyzable nitrogen, fast-acting phosphorus, fast-acting potassium, and organic matter were determined using the method of soil agrochemical analysis (3rd edition) by Bausch & Stang [25]. Soil pH and EC were measured using a soil–water ratio of 1:5. pH was determined using a pH meter (Five Easy Plus, Mettler Toledo, Greifensee, Switzerland); EC was determined using a conductivity meter (Environmental Express P200, Charleston, SC, USA); total soluble salts were determined using the mass method; salts and ions were determined using the flame photometric method and titration; hydrolytic nitrogen was determined using the alkaline dissolution and diffusion method; quick-acting carbon was determined using the alkaline dissolution method; quick-acting phosphorus was determined using the rapid phosphorus method. The diffusion method was used for hydrolytic nitrogen; the sodium bicarbonate leaching-molybdenum antimony colorimetric method was used for fast-acting phosphorus; the ammonium acetate leaching-flame photometric method was used for fast-acting potassium; and the oxidation of organic matter by potassium dichromate-external heating method was used. The photosynthetic indexes were measured via Yaxin-105 portable photosynthetic fluorometer (Beijing Yaxin Riyi Technology Co., Ltd., Beijing, China) from 12:00 to 14:00 under full sunlight. According to the instructions of the Angle Gene kit (Nanjing Aoqing Biotechnology Co., Ltd., Nanjing, China), 0.1 g of cotton leaf samples were collected and prepared in an ice bath using the internal extract of the kit. They were then tested for plant enzyme activity indicators, such as peroxidase (POD), catalase (CAT), nitrate reductase (NR), malondialdehyde (MDA), and superoxide dismutase (SOD). Roots were also collected. Root samples were cleaned with distilled water and dried on filter paper, and then prepared in an ice bath using the internal extraction solution of the kit to be tested for root vigor. At the end of the test, fresh soil samples were taken, dried at 37 °C, and sieved through a 30–50 mesh sieve. The samples were prepared according to the instructions of the Angle Gene kit to test for soil urease, nitrate reductase, alkaline phosphatase, acid phosphatase, ACP, β-glucosidase (S-β-GC), and soil catalase (S-CAT) and other soil enzymes. Plant enzyme activity, root vigor, and soil enzyme activity were measured via the micro method using an enzyme labeler (Thermo Scientific™ Multiskan™ GO full-wavelength enzyme labeler, Thermo Scientific, Gibco, Invitrogen, SIN, Waltham, MA, USA).

### 2.5. Statistical Analysis

Data were collated using WPS Office 2014, one-way analysis of variance (ANOVA) was performed using SPSS 26.0, R 4.3.2, and a Mantel test correlation between soil enzyme activity and physical and chemical properties was carried out using R. All the pictures in this study were plotted using R. Structural equation modeling data were made dimensionless using Matlab R2021b, and then standardized (normalized) using SPSS. The data were then standardized (normalized) using SPSS, and the structural equation model was constructed using the R programming language.

## 3. Results and Analyses

### 3.1. Changes in Soil Properties

Changes in soil pH during the different treatments in the different experimental cycles are shown in Table 1. Soil pH of the BC treatment alone was significantly higher than that of the CK one, increasing by 0.85–7.43% during the experimental period. It increased with the application rate but decreased over the course of the experimental period, showing a reduction of 12.60–15.20% at 90 days after seedling emergence compared to the levels at 30 days after seedling emergence of BC. Meanwhile, the pH of the WV treatments was significantly lower than that of the CK ones in all the treatments at 60 days after seedling emergence, which decreased by 12.00–24.00%. Compared with the CK, the water-soluble salt content in the single BC treatment increased significantly—BC1 and BC2 increased by 170.00% and 300.80%, respectively, while the WV treatment significantly reduced the soil water-soluble salt content, with WV1 and WV2 treatments decreasing by an average of 29.30% and 33.09%, compared to CK. The total exchangeable salts and sodium adsorption ratio followed the same trend as the changes in total water-soluble salt content.

**Figure 1:**
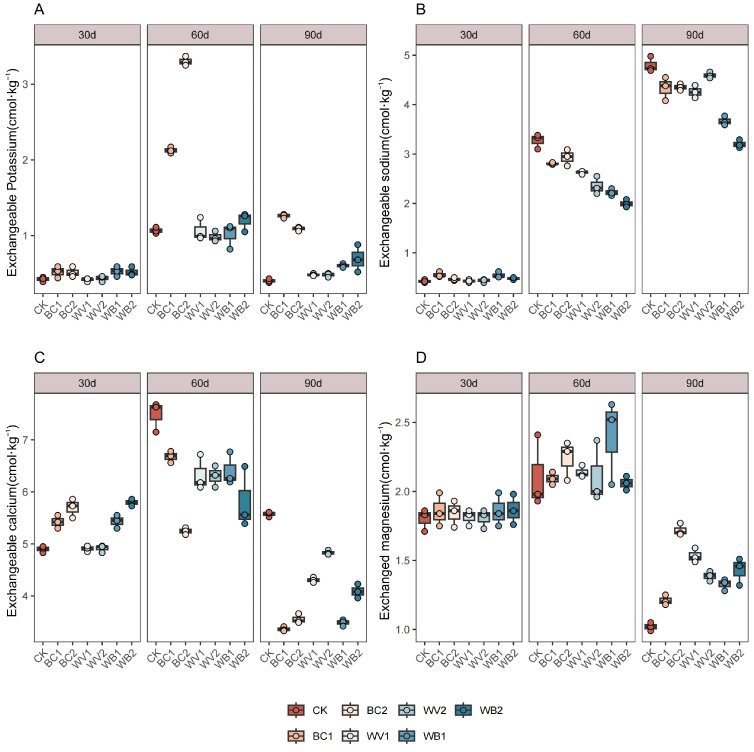
Effect of different treatments on exchangeable salt-based ions. Exchangeable potassium (**A**), Exchangeable sodium (**B**), Exchangeable calcium (**C**), Exchangeable magnesium (**D**). The length of the box shows the degree of dispersion of the data, the longer the length of the box, the more dispersed the data; the horizontal line in the box, representing the median, comparing the median size of multiple boxes can be roughly compared to the size of the data trend; up and down whiskers which is expressed as a line extending from the minimum to the maximum, which shows the box underneath and over the top (the minimum value) as well as underneath and above the upper (the maximum value) extends as far as the box.

The trend of soil exchangeable salinity ions of each treatment in different experimental cycles is shown in Figure 1. BC single application had the most significant effect on soil exchangeable potassium (Figure 1A). Compared with the CK treatment, BC1 and BC2 increased by an average of 111.60% and 133.70%. The exchangeable potassium ion content was the highest at 60 days after seedling emergence, and started to decrease at 90 days after seedling emergence, reducing by 53.39% compared to the levels at 60 days after seedling emergence. The WV treatment had little effect on the exchangeable potassium ion content. Exchangeable sodium ion content showed an overall increasing trend during the experimental period; compared with CK, WV and BC treatments reduced exchangeable sodium ion content (Figure 1B) by 4.31–28.03% and 9.31–14.17%, respectively. The reduction effect of their combined application was even more pronounced—compared with CK, WV, and BC alone, it decreased by 23.61–38.94%, 13.93–30.33% and 15.45–31.93%, respectively. At 30 days after the BC treatment, there was an increase in the exchangeable calcium ion content compared to the CK treatment (Figure 1C), with BC1 and BC2 increasing by 10.68% and 16.26%, respectively. After 60 days of the BC and WV treatments, there was a decrease in the exchangeable calcium ion content, reducing by 10.82–39.65% and 13.28–22.73% compared with the CK treatment. The exchangeable calcium ion content of the WV treatment was greater than the BC treatment at 90 days after emergence. The effect of BC alone on the exchangeable magnesium ion content at 30 days after emergence was not obvious (Figure 1D); 60 days after emergence, both WV and BC, either alone or in combination, increased the exchangeable magnesium ion content compared with the CK treatment. The effect of BC was greater than that of the WV treatment, and the dose of BC increased the exchangeable magnesium ion content, which differed among the various dosages of WV.

### 3.2. Characteristics of Changes in Soil Nutrient Content

**Figure 2:**
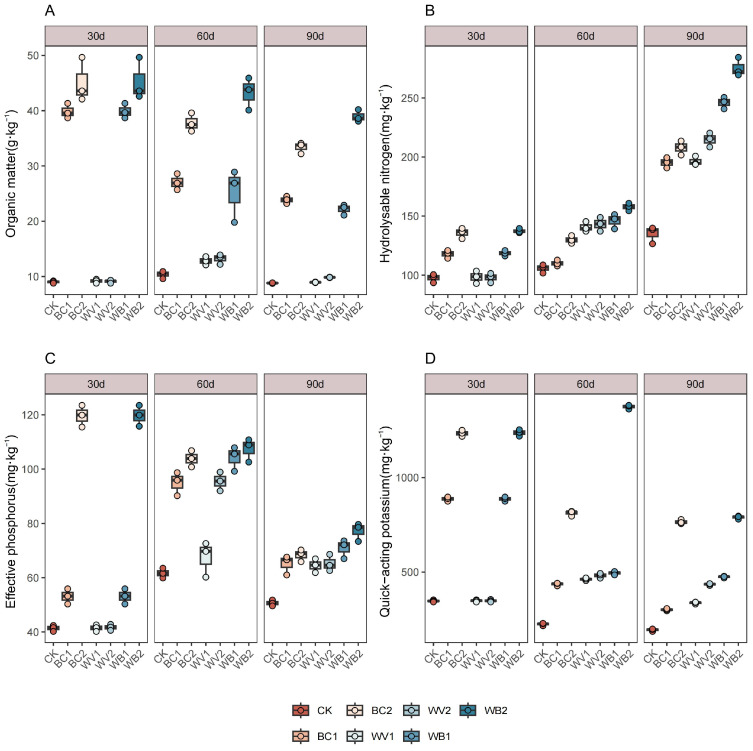
Effect of different treatments on soil nutrient content. Organic matter (**A**), Hydrolysable nitrogen (**B**), Effective phosphorus (**C**), Quick-acting potassium (**D**). The length of the box shows the degree of dispersion of the data, the longer the length of the box, the more dispersed the data; the horizontal line in the box, representing the median, comparing the median size of multiple boxes can be roughly compared to the size of the data trend; up and down whiskers which is expressed as a line extending from the minimum to the maximum, which shows the box underneath and over the top (the minimum value) as well as underneath and above the upper (the maximum value) extends as far as the box.

BC and WV applied alone or in combination increased soil nutrient content to different degrees (Figure 2). The BC application significantly increased soil organic matter content and gradually decreased with the continuation of the experimental period. At the beginning of the experiment, the highest organic matter content was found at 30 days after seedling emergence. Compared with the CK treatment, BC1 and BC2 increased by 343.00% and 401.00%. At the same time, over the course of the experimental period, WV was applied in combination with BC, resulting in the most significant increase in soil organic matter. With the increasing application rate, WB2 increased by 317.00–342.00% compared to CK during the experimental period. Hydrolysable nitrogen increased throughout the experimental period with BC and WV, both alone and in combination (Figure 2B). WB1 and WB2 increased by 82.20% and 104.00%, respectively, at 90 days after seedling emergence compared to CK. High-dose BC, applied alone or in conjunction with WV, showed a decreasing trend in soil effective phosphorus during the experimental period compared with CK. Thirty days after emergence, BC2 and WB2 increased by 188.97% and 189.21%, respectively, but by 90 days after emergence they decreased by 42.88% and 35.53% compared to 30 days after emergence, respectively. The low-dose BC1 applied alone and the WV treatment both showed a trend of increasing and then decreasing during the experimental period. The soil effective phosphorus content was the highest at 60 days after seedling emergence, with an increase of 78.67%, 62.78%, 128.83%, and 96.17% for BC1, WV1, WV2, and WB1, respectively, compared to 30 days after seedling emergence. Meanwhile, at 90 days after seedling emergence, the respective soil effective phosphorus levels decreased by 31.46%, 4.45%, 31.65%, and 31.99% compared to at 60 days. The changes in soil quick-acting potassium content in the different treatments were basically the same as those of effective phosphorus, but the WB2 treatment increased by 11.08% at 60 days after emergence compared to 30 days after emergence and then began to decrease.

### 3.3. Characteristics of Changes in Plant Morphological Indicators and Biomass

#### 3.3.1. Changes in Agronomic Indicators and Biomass of Plants

**Figure 3:**
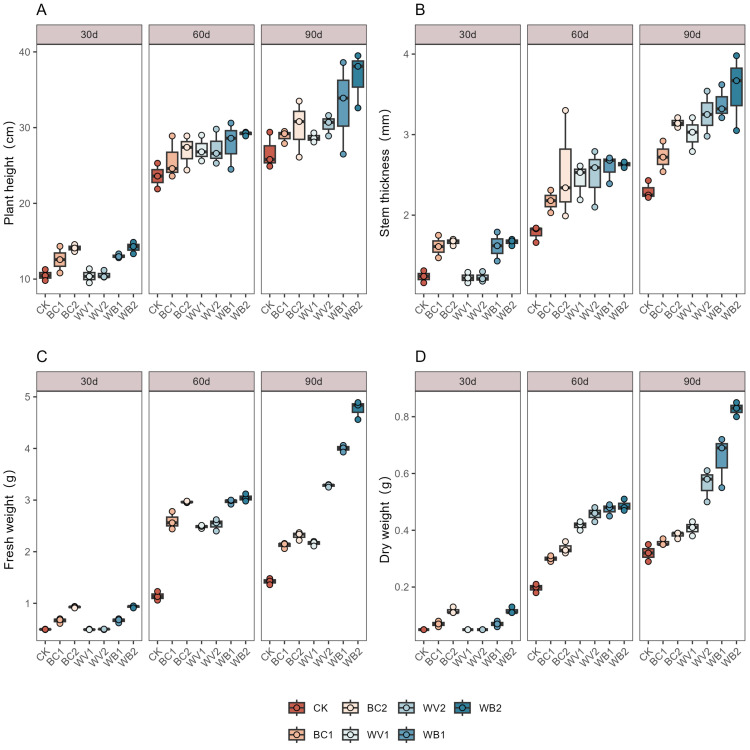
Effects of different treatments on the morphological indicators and biomass of plants. Plant height (**A**), Stem thickness (**B**), Fresh weight (**C**), Dry weight (**D**). The length of the box shows the degree of dispersion of the data, the longer the length of the box, the more dispersed the data; the horizontal line in the box, representing the median, comparing the median size of multiple boxes can be roughly compared to the size of the data trend; up and down whiskers which is expressed as a line extending from the minimum to the maximum, which shows the box underneath and over the top (the minimum value) as well as underneath and above the upper (the maximum value) extends as far as the box.

WV and BC applied alone or in combination promoted an increase in cotton plant height, stem thickness, fresh weight, and dry weight (Figure 3). The effect of the combined application was better than that of the single applications, with the effect of the combined application becoming more obvious throughout the experimental period. At the same time, with the increase in the dose of WV and BC, the promotional effect was gradually increased, combined application of WV and BC increased plant height by 14.32–21.90% at 90 d post emergence compared to WV and BC alone. stem thickness increased by 9.52–24.08%, fresh weight increased by 45.22–106.2%, and plant dry weight increased by 46.76–115.68%. At 60 days after seedling emergence, WV and BC applied alone did not instigate much change in terms of plant height, stem thickness, or fresh weight. At 90 days after seedling emergence, the effect of the WV treatment on stem thickness and on fresh weight was better than that of the BC treatment; compared with the BC treatment, stem thickness and fresh weight were increased by more than 3.50% and 1.88%, respectively. The effect of the WV treatment on the dry weight of the plants was also significantly higher than with the BC treatment (Figure 3D). Compared with the BC treatment, the dry weight of the plants increased by 14.02–46.96% more.

#### 3.3.2. Characteristics of Changes in Plant Photosynthetic Rate

**Figure 4:**
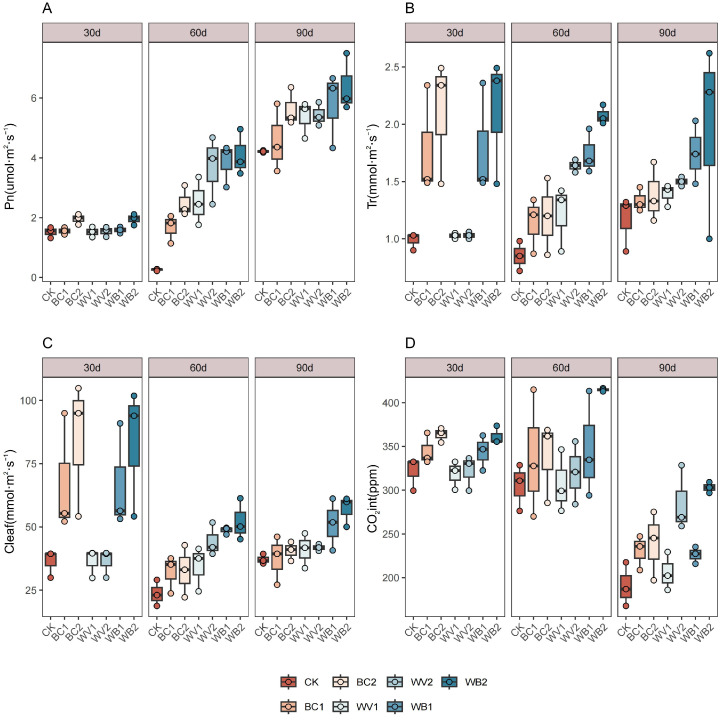
Effect of different treatments on photosynthetic characteristics of plants. Pphotosynthetic rate (Pn) (**A**), Transpiration (Tr) (**B**), Stomatal conductance (Cleaf) (**C**), Intercellular carbon dioxide concentration (CO_2_int) (**D**). The length of the box shows the degree of dispersion of the data, the longer the length of the box, the more dispersed the data; the horizontal line in the box, representing the median, comparing the median size of multiple boxes can be roughly compared to the size of the data trend; up and down whiskers which is expressed as a line extending from the minimum to the maximum, which shows the box underneath and over the top (the minimum value) as well as underneath and above the upper (the maximum value) extends as far as the box.

WV and BC applied alone or in combination increased the net photosynthetic rate (Pn) of the plants, which increased amount of the application. The combined application was better than when WV and BC were applied alone (Figure 4A). Ninety days after seedling emergence, the Pn of the WB2 treatments increased by more than 17.60% and 13.56% compared with that of WV and BC applied alone, and it continued to increase throughout the experimental period. Ninety days after seedling emergence, Pn increased by 177.91–263.86% compared with that at 30 days after seedling emergence, and Pn increased by 177.91–263.86% across treatments. Pn increased by 177.91–263.86% between treatments. WV and BC applied singly or in combination increased plant transpiration (Tr) and stomatal conductance (Cleaf), which increased with the application rate. The combined application was better than the single application (Figure 4B,C). During the experimental period, the WB treatments increased the transpiration (Tr) and stomatal conductance (Cleaf) compared to WV and BC by 23.30–72.85% and 5.47–72.85%, respectively. Between WV and BC treatments, WV was generally better than the BC treatment. There was no obvious regularity in the effect of WV and BC alone or in combination on the intercellular carbon dioxide concentration (CO_2_int) (Figure 4D), but, in general, the application of WV and BC increased the cotton CO_2_int compared to CK and increased with the amount of application.

### 3.4. Characteristics of Changes in Plant Enzyme Activities

**Figure 5:**
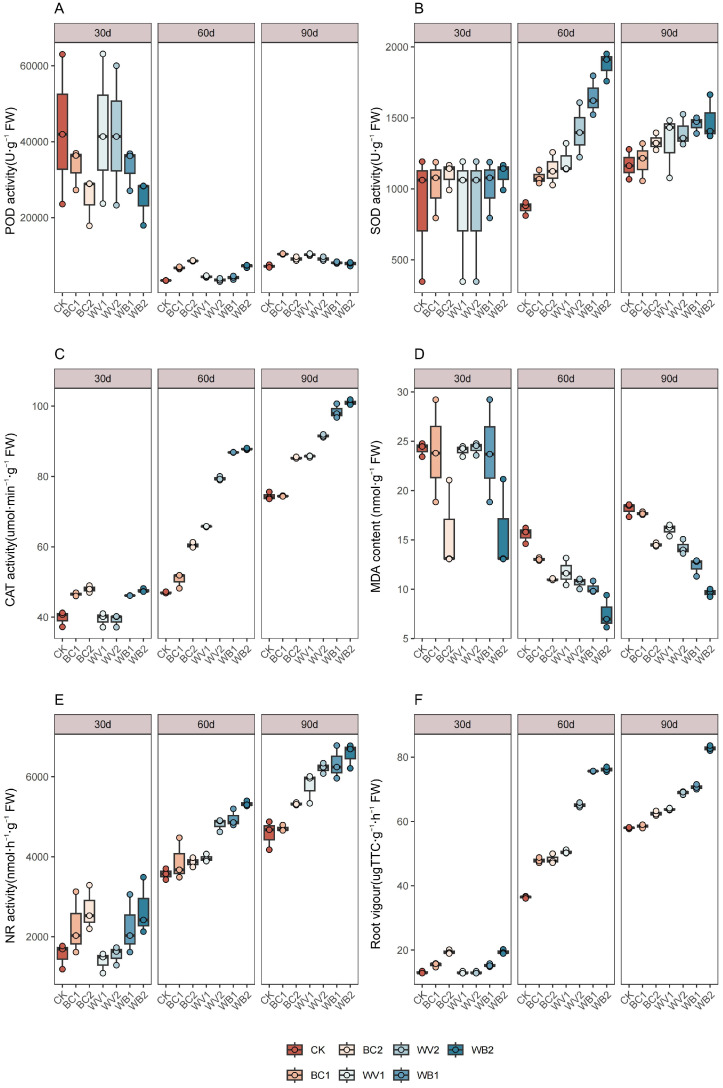
Effect of different treatments on the enzyme activities of plants. Peroxidase (POD) (**A**), Superoxide dismutase (SOD) (**B**), Catalase (CAT) (**C**), Malondialdehyde (MDA) (**D**), Nitrate reductase (NR) (**E**), Root vigour (**F**). The length of the box shows the degree of dispersion of the data, the longer the length of the box, the more dispersed the data; the horizontal line in the box, representing the median, comparing the median size of multiple boxes can be roughly compared to the size of the data trend; up and down whiskers which is expressed as a line extending from the minimum to the maximum, which shows the box underneath and over the top (the minimum value) as well as underneath and above the upper (the maximum value) extends as far as the box.

The effects of WV and BC alone or in combination on plant antioxidant enzyme activities are shown in Figure 5A–C. Peroxidase (POD) showed a general trend of decreasing and then increasing, and there was no obvious pattern of the effects of WV and BC alone or in combination on POD during the experimental period. BC and WV alone were better than the combination at 90 days after seedling emergence, and POD increased by 17.11–27.88% compared to the combination of BC and WV. WV and BC alone or in combination increased the plant superoxide dismutase (SOD) and catalase (CAT) activities, which increased with the increase in the application rate, and the combined application was better than the single application during the experimental period (Figure 5B,C). During the experimental period, the combined application of WV and BC increased SOD by 5.82–37.21% and 11.31–64.95%, and CAT by 10.36–32.05% and 18.43–71.40% compared with the single application of WV and BC, respectively. The effects of WV on SOD and CAT were greater than those of the BC treatment, and during the experimental period, WV increased SOD by 5.19–24.05% and CAT by 7.31–31.05% compared with BC, respectively. CAT increased by 7.31–31.35%.

WV and BC applied singly or in combination reduced the malondialdehyde (MDA) content of plants, and the combined application had a greater effect on MDA than the single application (Figure 5D). The decrease of MDA was more obvious with the increase in WV and BC application. During the experimental period, compared with CK, the MDA of WV and BC applied singly or in combination decreased by 2.47–51.72%. Compared with the WV and BC applied singly, the MDA of WV and BC applied in combination decreased by 13.64–32%, and the MDA of WV and BC treated singly decreased by 13.64–32%. The CAT increased by 7.31–31.35%. MDA decreased by 13.64–32.16% and 22.30–33.45%.

The changes in nitrate reductase (NR) in plants with different treatments are shown in Figure 5E. WV and BC applied alone or in combination increased NR activity, which increased with the increase of application rate, and the effect of the combined application was better than that of the single applications, increasing with the extension of the experimental period. During the experimental period, the NR of WV and BC applied in combination increased by 5.47–24.92 and 23.30–37.79% compared with that of WV and BC applied alone. The effect of WV on plant NR was greater than that of the BC treatment, with NR increasing by 2.16–22.55% compared with the BC treatment during the experimental period.

Changes in root vigor were shown in Figure 5F, WV and BC applied alone or in combination increased root vigor, which increased with the increase of application rate, and the effect of combined application was better than that of a single application, and increased with the extension of the experimental period; during the experimental period, the root vigor of WV and BC applied in combination increased by 10.83–49.69% and 20.87–57.99% compared with that of WV and BC applied alone; the role of WV on root vigor The effect of WV on root vigor was greater than that of BC treatment, and root vigor increased by 5.55–34.88% compared with BC treatment during the experimental period.

### 3.5. Characterisation of Changes in Soil Enzyme Activities

**Figure 6:**
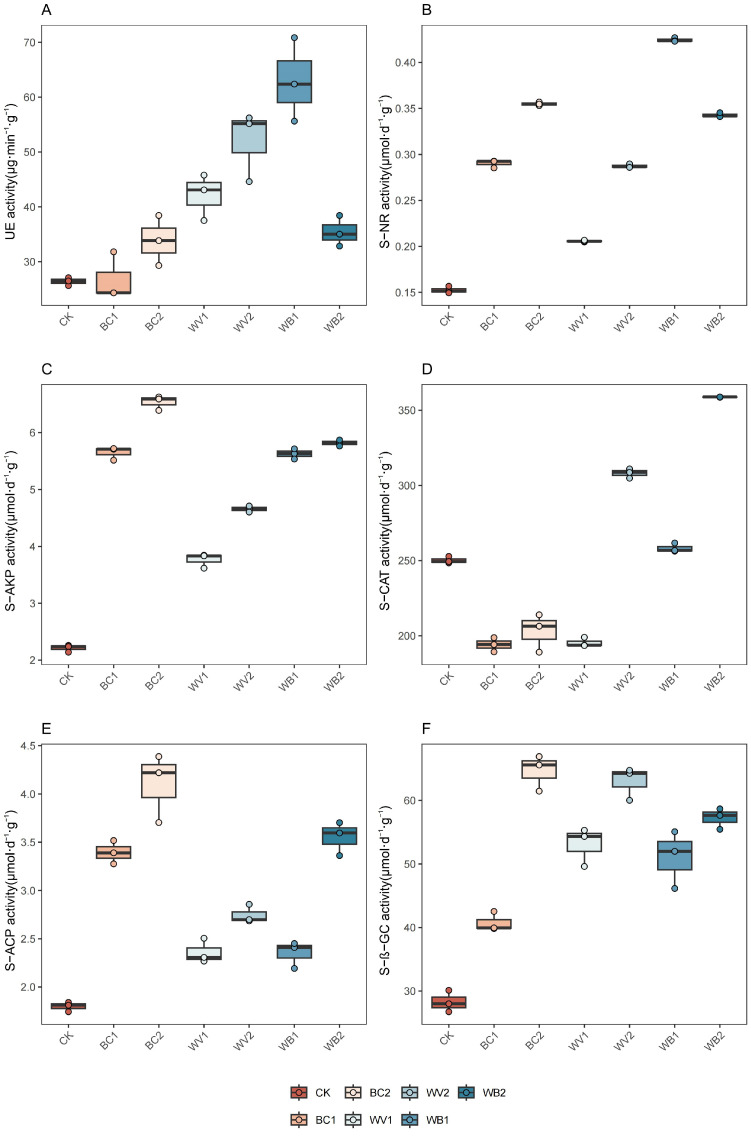
Effect of different treatments on soil enzyme activities. Soil urease (UE) (**A**), Soil nitrate reductase (S-NR) (**B**), Soil alkaline phosphatase (S-AKP) (**C**), Soil catalase (S-CAT) (**D**), Soil acid phosphatase (S-ACP) (**E**), Soil β-glucosidase (S-β-GC) (**F**). The length of the box shows the degree of dispersion of the data, the longer the length of the box, the more dispersed the data; the horizontal line in the box, representing the median, comparing the median size of multiple boxes can be roughly compared to the size of the data trend; up and down whiskers which is expressed as a line extending from the minimum to the maximum, which shows the box underneath and over the top (the minimum value) as well as underneath and above the upper (the maximum value) extends as far as the box.

The application of WV and BC alone or in combination increased soil urease (UE) content, which varied among treatments (Figure 6A). The low-dose BC1 treatment showed no significant change in UE, and the combined application with WV1 (WB1) significantly increased UE activity and was better than WV1 alone, with significant increases of 138.33%, 134.64%, and 49.40% compared to the CK, BC1, and WV1 treatments, respectively. The high-doses of BC2 and WV2 alone increased UE activity, with increases of 28.23% and 96.85% compared to the CK treatment, respectively, and their combined application reduced UE compared to WV2. The doses of BC2 and WV2 alone increased UE activity by 28.23% and 96.85% compared to the CK treatments, respectively, and their combined application decreased UE activity compared to WV2 but was still higher than in the CK treatments.

WV and BC alone or in combination increased soil nitrate reductase (S-NR), alkaline phosphatase (S-AKP), acid phosphatase (S-ACP), and β-glucosidase (S-β-GC) activities, which increased with increasing application rates (Figure 6B,C,E,F). The effects of BC alone or in combination with WV (except for the WB1 treatment of S-ACP) on the S-NR, S-AKP, S-ACP, and S-ACP activities of the treatment were higher than those of the WV monoapplication, with WV treatments reducing S-NR and S-AKP by 19.03–33.04% and 16.15–51.56% compared to BC and WB treatments. High-dose BC2 applied alone had the greatest effect on S-β-GC, increasing it by more than 7.87% compared to the BC1, WV, and WB treatments, while different doses of the WV and WB treatments had a greater effect on S-β-GC than low-dose BC1 treatments, increasing it by 20.19–35.31% compared to the BC1 treatments.

Changes in soil catalase (S-CAT) activity in different treatments are shown in Figure 6D. The BC and low-dose WV1 treatments decreased S-CAT activity. Compared to the CK treatment, BC1, BC2, and WV1 decreased by 22.44, 18.82, and 21.91%, respectively, while the high-dose WV2 alone and its combined application with high-dose BC increased S-CAT activity. Compared to CK treatment, WV2 and WB2 increased by 23.15% and 43.38%, respectively. The combined application of low-dose WV and BC had little effect on S-CAT.

### 3.6. Correlation Analysis

**Figure 7:**
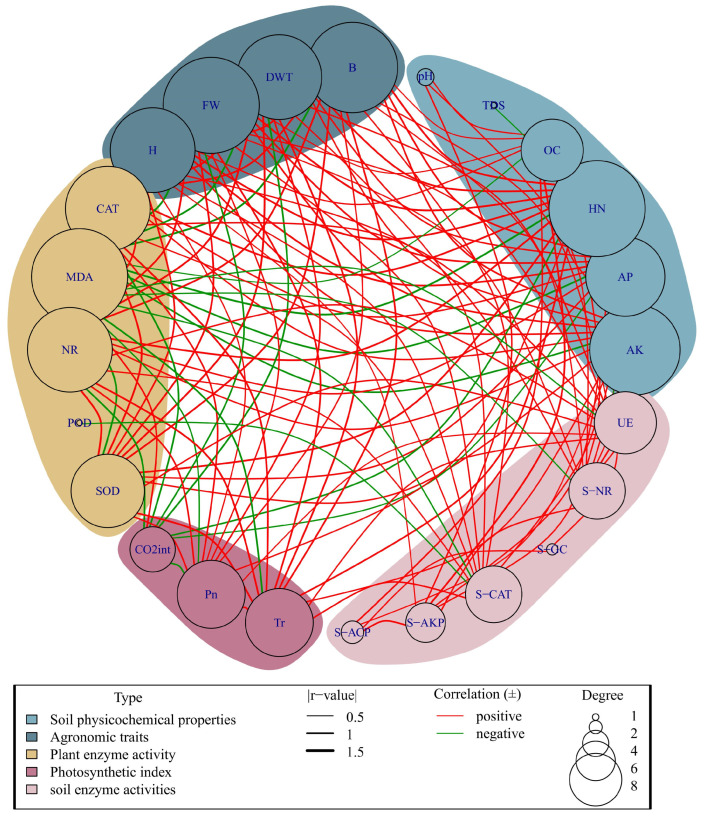
Results of Spearman correlation analysis (Correlation red line represents a positive correlation, green line represents a negative correlation. The thickness of the |r-value| line represents the correlation coefficient. pH, pH value; TDS, total dissolved solids; OC, organic matter; HN, hydrolysable nitrogen; AP, effective phosphorus; AK, quick-acting potassium; UE, soil urease; S-NR, soil nitrate reductase; S-GC, soil β-glucosidase; S-CAT, soil catalase; S-AKP, soil alkaline phosphatase; S-ACP, soil acid phosphatase; Tr, transpiration; Pn, net photosynthetic rate; CO_2_int, intercellular carbon dioxide concentration; SOD, plant superoxide dismutase; POD, plant peroxidase; NR, plant nitrate reductase; MDA, plant malondialdehyde; CAT, plant catalase; H, plant height; FW, fresh weight; DWT, dry weight; B, stem thickness).

The results of a Spearman correlation analysis of plant and soil indicators (Figure 7) showed that soil pH was significantly and positively correlated with soil alkaline phosphatase (*p* < 0.05, r = 0.785). Hydrolyzable nitrogen was positively correlated with plant morphology indicators (plant height, stem thickness, dry weight, and fresh weight) (r = 0.964, 1.000, 1.000, 0.964), and negatively correlated with MDA (r = −1.000). In addition, the lipid peroxidation of MDA was able to respond to the degree of oxidative stress and the degree of damage caused by adversity, and it was negatively correlated with plant morphology indices, soil nutrient content, and plant enzyme activities (CAT, NR, and SOD), which, to a certain extent, also indicated that the application of BC and WV could enhance the plant’s resistance to adversity. A positive correlation was found between CAT, NR, and SOD and the net photosynthetic rate of the plant (r = 0.857). A positive correlation was also found between the whole plant and the net photosynthetic rate of the plant. (r = 0.857). Soil nutrients showed strong correlations with plant morphology and enzyme activities in the whole analysis, while soil enzyme activities were not directly correlated with plant morphology and plant dry weight.

## 4. Discussion

### 4.1. BC and WV Application Affect Soil Properties

The results of this study showed that the application of BC alone increased soil pH and water-soluble salt content, and that WV application alone or in combination with BC decreased soil pH and water-soluble salt content. The significant increase in pH with BC alone was mainly due to the fact that soil exchangeable sodium was easily hydrated to form NaOH, which increased the alkalinity of the soil. CO_3_^2−^ and HCO_3_^−^ hydrolyzed to capture H^+^ from H_2_O and produced OH^−^ [26], resulting in an increase in soil pH. At the same time, due to the high pH characteristics of BC itself, it increased the soil pH. The content of organic acid in WV can be up to 11%, and the pH is 2.78. The soil pH for WV alone decreased significantly throughout the whole study and decreased with the increase in the application dose [27]. The joint application with BC can effectively avoid the increase of soil pH caused by the single application of BC. Moreover, WV and BC can effectively avoid the increase of soil pH caused by the single application of BC. At the same time, the combined application of WV and BC can significantly bring into play the fact that WV contains a large number of organic active molecules [27] and reacts with the soil to form complexes [28], which reduces the higher salinity ions brought in by BC applied alone [29].

### 4.2. BC and WV Application Affect Soil Nutrient Content

The combined application of BC and WV significantly increased the soil organic matter content. When combined with WV, the high organic carbon content of BC improves the ability to promote the formation of soil aggregate structure, creating a synergistic effect [30]. However, due to the decomposition and mineralization of active organic carbon fractions in BC [31], there was a noticeable decline in organic matter content in the later stages of the experiment. Both BC and WV, whether applied individually or in combination, increased the available nitrogen and effective phosphorus content in the soil. The combined application was more effective than the individual applications because BC provides a direct nutrient source of nitrogen and phosphorus [32]. Furthermore, the organic acids present in WV facilitate nitrogen release and convert the highly stable soil phosphorus into active phosphorus. This process enhances microbial activity and accelerates the release of phosphorus from biochar [14], thereby increasing the soil’s hydrolysable nitrogen and effective phosphorus content [33].

The available potassium ion content initially increased but then decreased throughout the experimental period. This trend can be attributed to the negatively charged functional groups (carboxyl and phenol groups) on the surface of biochar, which facilitate the adsorption of cations such as K^+^ [34]. This adsorption resulted in a significant increase in water-soluble and exchangeable potassium in the soil. Some studies have shown that biochar facilitates the conversion of non-exchangeable potassium to effective potassium, leading to a rapid release of water-soluble potassium shortly after application [35,36,37]. Elevated soil potassium content helps reduce Na+ uptake by plants [38], thereby mitigating the damage caused by salinity stress.

### 4.3. BC and WV Application Affected Plant Morphological Photosynthetic Indexes and Biomass

The application of BC and WV individually resulted in a significant increase in plant morphological indices and plant dry weight. Moreover, when WV and BC were applied together, the effect was even better than that of a single application. This can be attributed to the strong adsorption of BC, particularly for nitrogen, which prolongs the nutrient supply [39]. WV contains beneficial elements and organic acids that can regulate soil physicochemical properties and microbial communities, creating a favorable soil environment for plant growth [40]. Combining the two can maximize the benefits of both, promoting plant morphology, photosynthetic indices, and biomass [41]. Studies have shown that WV and BC can positively affect plant growth and yield, and the addition of BC can increase the biomass allocation of bolls, cottonseed, and lint in plants under salt stress, and increase the harvest index and water use efficiency [42]. Meanwhile, WV also showed different positive effects in terms of yield increase. Zhang Kangkang et al. [43] indicated that seed initiation treatment with a 2% wood vinegar solution significantly increased seed germination and seedling vigor of the rice variety ‘Drought Superior 73’, and promoted seedling dry matter accumulation [44]. The combined application of BC and WV to improve crop photosynthesis was superior to the single application of WV and BC The main reason is that BC improves photosynthesis by regulating the sodium/potassium homeostasis in plant cells and increasing the activities of CAT, NR, and SOD to reduce oxidative damage and damage caused by adversity [45]; WV can produce physiological activity in crops through its phenolic and organic acids [46], increasing the stomatal conductance of leaves and the concentration of carbon dioxide entering the stomata to ensure the supply of photosynthesis to the plants. The supply of photosynthesis is enhanced by BC, which contains calcium, magnesium, and other minerals necessary for crop growth, providing the required trace elements [47]. The joint application of the two can be enough to better promote the release of BC and soil nutrients, which affects the plant morphology, photosynthesis indicators, and biomass.

### 4.4. BC and WV Applications Affect Plant Enzyme Activities

The single application of BC showed that plant peroxidase (POD) exhibited an initial decline and then an increasing trend. This may be due to the higher salt content of BC itself, which, in the initial period of application, increases soil salinity. Salt stress can lead to the production of reactive oxygen species damage that intracellular proteins and unsaturated fatty acids, resulting in lipid peroxidation of the cell membrane [48]. As the experimental period continued, both the aging of BC and its combined application with WV, which contains phenols, could stimulate the plant’s endogenous growth hormone and a variety of enzymes [49]. This promotes an increase in POD, CAT and SOD, which are the main antioxidant enzymes that can synergistically scavenge ROS [50]. They also play an important role in preventing the formation of free radicals, scavenging free radicals, and delaying plant senescence, etc. In this study, superoxide dismutase (SOD) increased and then decreased, while catalase (CAT) showed an increasing trend. This is because BC can promote the production of more antioxidant enzymes within the plant, promote the secretion of key enzymes, and play a detoxifying role in cellular peroxides [51]. The joint application of WV and BC can stimulate the plant to activate its defense mechanism of producing defensive substances in response to the acidity in WV. The activity of SOD catalyzes the scavenging of O_2_ radicals to form H_2_O_2_, and CAT then changes H_2_O_2_ into H_2_O. SOD and CAT are the main catalysts for the formation of H_2_O_2_ into H_2_O. Together, SOD and CAT coordinate with each other to reduce the toxicity of free radicals and keep free radicals and reactive oxygen species at relatively low levels [52,53], improving plant resistance.

Malondialdehyde (MDA) is one of the most important products of membrane lipid peroxidation, and its production can exacerbate membrane damage. In this study, the overall MDA of BC and WV applied singly or in combination showed a decreasing trend to different degrees, with the decreasing trend of MDA in the combined application being greater than that of the single applications. This was attributed to the fact that the combined application of BC and WV could give fully utilize the protective effect of BC-rich, stabilized carbon-containing organic compounds in plants, resisting the infiltration of exotic pathogens and protecting the cells from infestation [54]. Additionally, the plant’s absorption and utilization of the small and medium molecule organic acids from WV promote photosynthesis, increase the photosynthetic rate, and slow down the aging rate of crops [49]. This achieves the dual role of inhibiting both the accumulation of plant MDA and the occurrence of membrane lipid peroxidation—maintaining intracellular ionic balance—and ensuring a variety of metabolic processes are carried out normally [55].

BC and WV applied alone or in combination increased nitrate reductase (NR) in plants, and the increase was more significant in the combined application. This is because, in addition to its nutrient supply, the combined application of WV and BC can also fully utilize the absorption and slow release of soil fertilizers by BC, improve the nutrient supply capacity of the soil, and alleviate the inhibitory effect of saline and alkaline stresses on the activity of enzymes related to leaf nitrogen metabolism [56]. At the same time, it can exhibit WV’s growth hormone-like effects to promote the rate of plant N metabolism and improve plant NR activity [57].

Combined application of BC and WV can more significantly improve cotton root vigor compared with a separate application. The combined application of BC and WV makes the alkalinity of BC and the acidity of WV improve the soil environment of the root system through acid-base neutralization [58]. This process promotes the release of soil nutrients and mineral transformation and improves the biological properties of the soil [59], which in turn alters the structure of the root system, promotes the growth of roots, and improves the activity of the root system [58].

### 4.5. Application of BC and WV Affects Soil Enzyme Activities

Soil enzyme activity directly reflects the intensity of soil biochemical reactions, and soil urease (UE) is mainly derived from enzymes released from the decomposition of plant and animal residues and secretions from soil microorganisms and plant roots [60]. Urease promotes the decomposition of urea and can characterize soil nitrogen availability [61]. The results of this study showed that UE tended to increase and then decrease in different treatments. WV alone and low-dose WV combined with BC were better than BC alone and high-dose WV combined with BC, and all treatments were better than CK. This may be due to the nitrogen content of BC itself and to the trace elements in WV, which promote the conversion of nitrogen in the soil [61], leading to an increase in UE. BC and WV alone or combined increased nitrate reductase (S-NR), and the optimal effect of the combined application was mainly because the combined application of WV and BC was able to promote the release of BC’s carbon source, which promoted microbial reproduction, thus further stimulating S-NR [62,63]. Alkaline phosphatase (S-AKP) and acid phosphatase (S-ACP) tended to increase in different treatments, with a significant increase in S-AKP in BC alone and the co-application with WV, and higher S-ACP in the high-dose BC treatment than in the low-dose BC alone as well as in WV alone or in the co-application with the low-dose BC, mainly because of the alkaline nature of the BC in the supply. It was found that application of BC S-AKP enhanced activity more significantly than that of S-ACP (18.9% and 3.3%), and that higher phosphatase activity increased phosphorus effectiveness [64]. A Spearman’s correlation analysis showed that organic matter was highly significantly and positively correlated with S-AKP (*p* < 0.01, r = 0.964) and with S-ACP (*p* < 0.05, r = 0.857). In addition, increased organic matter activates microbial functions and improves the catalytic efficiency of enzymes, thus increasing the bioavailability of nitrogen and phosphorus in the soil to sustain continuous microbial reproduction and metabolism and promote nutrient cycling [65]. The inconsistent trend of overall changes in catalase (S-CAT) may be attributed to the elevated soil salinity limiting microbial activities due to the higher salt content of BC itself [66,67], which led to a decrease in S-CAT in the BC treatment, whereas the high dose of WV alone or in combination with BC increased the S-CAT because WV application lowers the soil pH, improves the effective supply of nutrients, promotes microbial growth and reproduction, and increases microbial secretion of metabolites, resulting in increased soil enzyme activities [68]. β-Glucosidase (S-β-GC) showed optimal results in the BC2 treatment, S-β-GC was positively correlated with soil OC content, and biochar was found to improve the soil environment, increase the content of soil organic matter, and increase the related enzyme activities [69]. While the WB treatment decreased probably because WV promotes the nutrients adsorbed by BC to be released gradually, and the adsorption binding sites for soil enzymes increased, thus inhibiting the reaction of S-β-GC [70].

### 4.6. Comprehensive Evaluation Analysis of Different Treatments

Both WV and BC treatments showed improved plant agronomic traits, enzyme activity, and soil nutrients, and increased plant dry matter mass. Based in our comprehensive analysis of the indicators between different treatments of BC and WV (Table 2, entropy weighting), the best performance was achieved by the combined application of both in the WB2 treatment, followed by the WB1 treatment. The reason for this difference may be due to the change in soil physicochemical properties and the increase in plant nutrient utilization efficiency, which enhances its stress tolerance and nutrient content.

## 5. Conclusions

WV and BC, whether applied alone or in combination, have been found to enhance soil nutrients, soil enzyme activity, and plant photosynthesis, thereby promoting crop growth. Interestingly, WV alone has shown greater effectiveness compared to BC alone, while the combined application of WV and BC has proven to be even more effective than using each of them separately. Furthermore, it has been observed that increasing the dosage of the application enhances its effectiveness. Soil enzyme activity is an indicator that is responsive to changes in the soil environment. Our findings demonstrate a highly significant positive correlation between soil enzyme activity and soil nutrient content. Consequently, future research should focus on strengthening the investigation of the early-stage impact of WV and BC on soil enzyme activity, particularly in relation to the improvement of saline and alkaline soils. Moreover, attention should be directed towards studying the interplay between WV, BC, soil enzyme activity, and soil antibiotic resistance genes (ARGs) to mitigate the adverse effects of antibiotics, excessive fertilizer use, and pesticide application on soil health.

## Figures and Tables

**Table 1:** Effect of different treatments on soil properties.

Test Cycle	Treatment No.	pH	Total Water-Soluble Salt (g·kg^−1^)	Conductivity (mS/cm)	Total Exchangeable Salts (cmol·kg^−1^)	Sodium Adsorption Ratio
30 days	CK	9.15 ± 0.01 c	1.30 ± 0.24 c	0.15 ± 0.06 c	7.55 ± 0.11 b	1.38 ± 0.14 b
	BC1	9.40 ± 0.04 b	3.51 ± 0.11 b	1.01 ± 0.02 b	8.41 ± 0.23 a	7.55 ± 0.41 a
	BC2	9.83 ± 0.06 a	5.21 ± 0.24 a	1.60 ± 0.02 a	8.52 ± 0.20 a	8.91 ± 0.98 a
	WV1	9.15 ± 0.01 c	1.32 ± 0.25 c	0.15 ± 0.05 c	7.54 ± 0.10 b	1.38 ± 0.13 b
	WV2	9.15 ± 0.01 c	1.29 ± 0.24 c	0.14 ± 0.04 c	7.55 ± 0.11 b	1.38 ± 0.14 b
	WB1	9.41 ± 0.04 b	3.54 ± 0.14 b	1.08 ± 0.07 b	8.41 ± 0.23 a	7.53 ± 0.40 a
	WB2	9.86 ± 0.04 a	5.25 ± 0.22 a	1.60 ± 0.02 a	8.53 ± 0.19 a	8.95 ± 1.00 a
60 days	CK	8.28 ± 0.05 b	9.11 ± 0.01 a	2.31 ± 0.01 a	12.87 ± 0.57 g	11.04 ± 0.15 a
	BC1	8.35 ± 0.02 b	6.35 ± 0.07 c	1.98 ± 0.01 b	15.87 ± 0.57 f	9.70 ± 0.15 b
	BC2	8.54 ± 0.02 a	7.34 ± 0.09 b	1.87 ± 0.01 c	18.87 ± 0.57 e	9.83 ± 0.09 b
	WV1	7.32 ± 0.02 e	6.20 ± 0.04 c	1.68 ± 0.01 d	21.87 ± 0.57 d	9.50 ± 0.23 b
	WV2	6.27 ± 0.03 f	5.71 ± 0.02 d	1.50 ± 0.01 e	24.87 ± 0.57 c	9.27 ± 0.23 b
	WB1	8.06 ± 0.03 c	4.51 ± 0.06 e	0.90 ± 0.04 g	27.87 ± 0.57 b	9.15 ± 0.79 b
	WB2	7.83 ± 0.03 d	5.90 ± 0.09 d	1.28 ± 0.01 f	30.87 ± 0.57 a	9.30 ± 0.03 b
90 days	CK	8.00 ± 0.02 e	9.98 ± 0.03 a	2.85 ± 0.01 a	13.93 ± 0.12 a	23.17 ± 5.90 a
	BC1	8.22 ± 0.01 b	6.73 ± 0.09 e	1.95 ± 0.01 c	13.70 ± 0.03 a	10.76 ± 0.16 b
	BC2	8.34 ± 0.01 a	7.64 ± 0.02 c	1.72 ± 0.01 d	13.72 ± 0.10 a	12.01 ± 0.75 b
	WV1	8.09 ± 0.01 d	7.32 ± 0.02 d	1.64 ± 0.01 d	12.10 ± 0.30 b	10.32 ± 0.14 b
	WV2	6.31 ± 0.04 f	7.10 ± 0.03 d	1.64 ± 0.01 d	11.75 ± 0.38 bc	9.43 ± 0.13 b
	WB1	8.18 ± 0.02 bc	6.49 ± 0.07 e	1.75 ± 0.01 d	12.04 ± 0.08 b	8.70 ± 0.14 b
	WB2	8.11 ± 0.01 cd	8.71 ± 0.17 b	2.15 ± 0.10 b	11.06 ± 0.28 c	9.60 ± 0.36 b

Note: different lowercase letters indicate significant differences at the 0.05 probability level (*p* < 0.05), determined by one-way analysis of variance (ANOVA) and Duncan’s post hoc test. The data are presented as means ± standard deviation (SD) calculated from three repetitions.

**Table 2:** Comprehensive evaluation analysis of the different treatments.

Index Value	Positive Ideal Solution	Negative Ideal Solution	Comprehensive Score	Sort
CK	0.918	0.327	0.262	7
BC1	0.795	0.383	0.325	6
BC2	0.645	0.531	0.451	4
WV1	0.735	0.378	0.339	5
WV2	0.542	0.597	0.524	3
WB1	0.525	0.64	0.549	2
WB2	0.455	0.831	0.646	1

Note: This table uses the entropy weighting method for a comprehensive evaluation, and the data of all indicators are quantified and normalized for data analysis.

## Data Availability

Data are contained within the article.
